# Bidirectional Association between Sarcopenia and Depressive Symptoms among Chinese Middle- and Older-Aged Adults: Longitudinal Observational Study

**DOI:** 10.3390/brainsci14060593

**Published:** 2024-06-11

**Authors:** Na Zeng, Chao Li, Huan Mei, Shuilin Wu, Chang Liu, Xiaokun Wang, Jie Shi, Lin Lu, Yanping Bao

**Affiliations:** 1 School of Public Health, Peking University, Beijing 100191, China; zengna19@bjmu.edu.cn (N.Z.); meihuan@pku.edu.cn (H.M.); 2211210073@stu.pku.edu.cn (S.W.); liuchang1@stu.pku.edu.cn (C.L.); 2311110171@bjmu.edu.cn (X.W.); 2 National Institute on Drug Dependence and Beijing Key Laboratory of Drug Dependence, Peking University, Beijing 100191, China; shijie@bjmu.edu.cn; 3 Beijing Friendship Hospital, Capital Medical University, Beijing 100050, China; lichao2988@mail.ccmu.edu.cn; 4 National Clinical Research Center for Mental Disorders, Key of Mental Health, Ministry of Health (Peking University), Peking University Institute of Mental Health, Peking University Sixth Hospital, Beijing 100191, China; 5 Peking-Tsinghua Centre for Life Sciences and PKU-IDG/McGovern Institute for Brain Research, Peking University, Beijing 100191, China

**Keywords:** sarcopenia, possible sarcopenia, depressive symptoms, bidirectional relationship

## Abstract

Background: The study aimed to examine the bidirectional relationship between sarcopenia and depressive symptoms in a national, community-based cohort study, despite the unclear temporal sequence demonstrated previously. Methods: Data were derived from four waves (2011 baseline and 2013, 2015, and 2018 follow-ups) of the China Health and Retirement Longitudinal Study (CHARLS). A total of 17,708 participants aged 45 years or older who had baseline data on both sarcopenia status and depressive symptoms in 2011 were included in the study. For the two cohort analyses, a total of 8092 adults without depressive symptoms and 11,292 participants without sarcopenia in 2011 were included. Sarcopenia status was defined according to the Asian Working Group for Sarcopenia 2019 (AWGS 2019) criteria. Depressive symptoms were defined as a score of 20 or higher on the 10-item Center for Epidemiologic Studies Depressive Scale (CES-D-10). Cox proportional hazard regression models were conducted to examine the risk of depressive symptoms and sarcopenia risk, while cross-lagged panel models were used to examine the temporal sequence between depressive symptoms and sarcopenia over time. Results: During a total of 48,305.1 person-years follow-up, 1262 cases of incident depressive symptoms were identified. Sarcopenia exhibited a dose–response relationship with a higher risk of depressive symptoms (HR = 1.7, 95%CI: 1.2–2.3 for sarcopenia, and HR = 1.5, 95%CI: 1.2–1.8 for possible sarcopenia, *p* trend < 0.001). In the second cohort analysis, 240 incident sarcopenia cases were identified over 39,621.1 person-years. Depressive symptoms (HR = 1.5, 95%CI: 1.2–2.0) are significantly associated with a higher risk of developing sarcopenia after multivariable adjustment (*p* < 0.001, Cross-lagged panel analyses demonstrated that depressive symptoms were associated with subsequent sarcopenia (β = 0.003, *p* < 0.001). Simultaneously, baseline sarcopenia was also associated with subsequent depressive symptoms (β = 0.428, *p* < 0.001). Conclusion: This study identified a bidirectional relationship between depressive symptoms and sarcopenia. It seems more probable that baseline sarcopenia is associated with subsequent depressive symptoms in a stronger pattern than the reverse pathway. The interlinkage indicated that maintaining normal muscle mass and strength may serve as a crucial intervention strategy for alleviating mood disorders.

## 1. Introduction

Sarcopenia and depressive symptoms are two major contributors to disease burden worldwide, limiting functional abilities and diminishing quality of life in the elderly [1,2,3,4]. Both conditions were common, with major depressive disorder having a life-time prevalence of an estimated 15.0–18.0% and 4.6–9.3% in the elderly population [5,6,7,8]. Conversely, the prevalence of sarcopenia ranged from 8% to 36% in middle-aged and 11.5% to 40% in older adults [9,10,11,12]. Both depressive symptoms and sarcopenia are associated with a substantial risk of negative outcomes, resulting in personal suffering, family disruption, and imposing a substantial economic burden on society [13,14,15]. Previous studies have reported that these two conditions have the potential to engender significant health burdens and considerable adverse consequences in conjunction with the global aging population trend [16,17].

Irrespective of temporality, the co-occurrence of sarcopenia and depressive symptoms is common [17,18]. Overall, 28% of sarcopenia patients had depressive symptoms, while 32.8% of patients with depressive symptoms had comorbid sarcopenia [18,19]. Sarcopenia is associated with an 80% higher risk of depressive symptoms, and depressive symptoms increased sarcopenia risk by 24–113% [17,18,20]. Emerging evidence supports a possible association between sarcopenia and depressive symptoms due to the shared plausible pathogenetic mechanisms, including physical inactivity, the upregulation of inflammatory factors (i.e., elevated level of interleukin (IL)-6, C-reactive protein (CRP), and tumor necrosis factor-alpha (TNF-alpha)), the alteration of gut microbiota composition, insulin resistance (IR), and hormonal imbalances in the hypothalamic–pituitary–adrenal axis [17,18,21,22,23].

According to previous studies, adults with sarcopenia and depressive symptoms had lower cognitive performance [24,25] and physical activity levels [26,27] and higher risk of falls [28,29] than those without these conditions. Other studies revealed that older adults with both sarcopenia and depressive symptoms had a higher risk of falls and hospitalization than those without these conditions [18]. Additionally, it has been reported that both sarcopenia and depressive symptoms serve as risk factors for cardiovascular disease and are linked to long-term mortality associated with it [30,31,32]. In summary, the co-occurrence of the two conditions results in impaired functioning and incapacity, which diminishes overall well-being and raises the risk of illness and death.

However, while the majority of previous studies primarily focused on establishing a correlation between sarcopenia and depressive symptoms using cross-sectional data [19,33], more recent longitudinal studies have begun to explore the prospective effects of depression on sarcopenia. Nevertheless, the bidirectional and temporal sequence between sarcopenia and depressive symptoms remains unclear. Understanding the potential temporal sequence between sarcopenia and depressive symptoms is crucial for early detection and intervention, ultimately facilitating effective disease management and improving overall quality of life. Thus, to address these knowledge gaps, our study aimed to investigate the bidirectional association between sarcopenia and depressive symptoms using data from a large Chinese national community-based prospective cohort study with an extensive long-term follow-up.

## 2. Methods

### 2.1. Study Population

All of data in this study were collected from the 2011, 2013, 2015, and 2018 waves of the China Health and Retirement Longitudinal Study (CHARLS), including the assessment of depression and sarcopenia. CHARLS constitutes a continuous survey program characterized by periodic examinations recurring at intervals of 2 to 3 years. Within this framework, participants undergo face-to-face interviews within the confines of their own residences, facilitated by the deployment of computer-assisted personal interviewing (CAPI) technology. Detailed information regarding the study design of the CHARLS have been previously reported [34]. The Institute of Social Science Survey at Peking University maintains all CHARLS-collected data, which has been made available to the public via the CHARLS website (http://charls.pku.edu.cn/en, accessed on 27 May 2023).

In this present study, we analyzed data from the CHARLS 2011, 2013, 2015, and 2018. A total of 17,708 respondents were recruited in the 2011 survey. The inclusion criteria for the cohort 1 (cohort for the onset of depressive symptoms) were as follows: (1) individuals who were 45 years of age or older in 2011; (2) individuals who had baseline data available for assessing sarcopenia status; (3) individuals who neither received a diagnosis of mood disorders from medical professionals nor took any medication for addressing emotional, nervous, or mental issues; and (4) those who obtained CES-D-10 scores ≤ 10 in 2011. The exclusion criteria were as follows: (1) individuals who did not engage in the assessment of depressive symptoms in 2013, 2015, or 2018; and (2) individuals with missing data on all demographic characteristics and confounders. Ultimately, 8092 participants were included in the first cohort analysis. For cohort 2, focused on the onset of sarcopenia, inclusion criteria were (1) individuals aged 45 years or older in 2011; and (2) individuals who had baseline data on the assessment of depressive symptoms in 2011, either through participation in the CES-D-10 evaluation or by reporting a diagnosis of mood disorders by doctors or taking medication for mood disorders. The exclusion criteria comprised the following: (1) absence of data pertaining to all components associated with sarcopenia in the years 2013, 2015, or 2018; (2) participants who already had sarcopenia at baseline; (3) lack of information concerning demographic variables and covariates. Finally, 11,292 participants were included in the second cohort analysis (Table A1). Within cohort 1, approximately half of the participants were female (3717/8092, 45.93%), with a median age of 57.0 years. Cohort 2 comprised 47.33% females (5345/11,292), with a median age of 55.0 years.

### 2.2. Assessment of Sarcopenia

The assessment of sarcopenia was conducted in accordance with the 2019 Asian Working Group for Sarcopenia (AWGS) criteria, which included evaluations of physical performance, appendicular skeletal muscle mass (ASM), and muscle strength. The hands of both the dominant and non-dominant individuals were assessed for handgrip strength, which was quantified in kilograms, using a Yuejian^TM^ WL-1000 dynamometer (Nantong, China). Participants were instructed to exert maximal force while squeezing the dynamometer, with two measurements taken for each hand while holding the dynamometer at a right angle (90°). In analyses, the mean value of the maximal strength data was utilized. When measurement of one hand was impossible because of any reason, the greatest score obtained from the other hand was documented. Low handgrip strength was defined as less than 28 kg for males and less than 18 kg for females, in accordance with the AWGS 2019 criteria. A validated anthropometric equation was employed to estimate the ASM of the Chinese population, as reported by Chinese residents [30,35].

The weight and height of the subjects were determined utilizing an Omron^TM^ HN-286 scale (Kyoto, Japan) and a Seca^TM^213 height meter (Hamburg, Germany), respectively. A former study has illustrated that the ASM computed through this formula strongly correlates with measurements derived from dual-energy X-ray absorptiometry (DXA) [36]. The threshold for defining low muscle mass was determined by identifying the sex-specific lowest 20th percentile of height-adjusted muscle mass (ASM/height^2^) within the study cohort, with values of <5.29 kg/m^2^ for women and <6.99 kg/m^2^ for men, consistent with previous studies [37].

Physical performance evaluation encompassed measures of gait speed, the five-time chair stand test, and the Short Physical Performance Battery (SPPB) [38]. In order to evaluate gait speed, participants were directed to walk a distance of 2.5 m at their typical speed, and their durations of completion were documented over the course of two trials. The five-time chair stand test documented the amount of time necessary to ascend consecutively from a chair with a cross-chest fold, measuring 47 cm in height. The SPPB assessment consisted of three balance tests, each lasting 10 s: (1) side-by-side stance; (2) semi-tandem stance (with the heel of one foot placed next to the big toe of the other foot); and (3) tandem stance (with the heel of one foot contacting the toes of the other foot). Each person’s examination was given a maximum of four points, resulting in a cumulative score of twelve. Per the AWGS 2019 guidelines, low physical performance was characterized by a gait speed below 1.0 m/s, a five-time chair stand test taking 12 s or longer to complete, or an SPPB score falling below 9.

Possible sarcopenia can be identified by reduced muscular strength or poor physical performance. Sarcopenia is characterized by an accompanying reduction in muscle mass, strength, or physical performance. Consequently, participants were categorized into three groups: non-sarcopenia, possible sarcopenia, and sarcopenia.

### 2.3. Depressive Symptoms Ascertainment

This study assessed depressive symptoms using the 10-item Center for Epidemiologic Studies Depressive Scale (CES-D-10), which is a refined version derived from the original 20-item CES-D. Participants were queried about the frequency of experiencing ten listed symptoms during the preceding week. Responses were categorized as follows: rarely or never (<1 day), sometimes (1–2 days), occasionally (3–4 days), and most or all of the time (5–7 days). Each item was assigned a score from 0 (rarely) to 3 (most or all of the time), with items 5 and 8 reverse-scored before score summation. The total CES-D-10 score ranged from 0 to 30. Consistent with prior research, participants scoring 20 or higher were classified into the depressive symptoms group, those with a score of ≥10 were categorized similarly, while those scoring <10 were designated as the no depressive symptoms group. [39,40]. The CES-D-10 has undergone comprehensive validation for application in general populations and has exhibited sufficient levels of reliability and validity when applied to senior adults residing in the community in China [41].

### 2.4. Covariates

Covariates were chosen in consideration of prior epidemiological evidence and the availability of data at baseline [10,17,24,30]. Potential confounding variables encompassed age (continuous), sex (male and female), BMI (<25 kg/m^2^ and ≥25 kg/m^2^), residential location (urban and rural), educational attainment (from illiterate to primary school, middle school, and high school to college or higher enrolled), average household income (CNY <1000, 1000, 5000, 10,000, and 20,000), marital status (including married, separated, and unmarried/divorced/widowed), smoking status (including never, current, and previous), alcohol intake (including never, current, and previous), daily sleep time, presence of comorbid physical diseases (including cancer, chronic lung diseases, heart disease, stroke, arthritis, dyslipidaemia, hepatic disease, kidney disease, digestive system disease, asthma, and memory-related diseases such as dementia, brain atrophy, and Parkinson’s disease, as well as hypertension and diabetes), and cognitive function. The presence of comorbidities was assessed through the CHARLS questionnaire, which inquired about participants’ diagnosed conditions and treatments by asking, “Have you been diagnosed with this condition by a doctor?” and “Are you currently undergoing any treatment for this condition?” Participants who affirmed either having the condition or undergoing treatment were categorized as having the condition in our study. Cognitive function was assessed by Mini-Mental State Examination (MMSE) results [42].

### 2.5. Statistical Analysis

In order to summarize continuous variables, means (standard deviations (SDs)) or medians (interquartile range (IQR)) were utilized. Subsequently, to compare differences between groups, we conducted independent *t* tests, one-way analysis of variance (ANOVA) analyses, Wilcoxon tests, and Kruskal–Wallis tests. χ^2^ tests were performed to compare categorical or ordinal variables represented as numbers (proportions). The cumulative incidence rate was determined using the Kaplan–Meier method. Subsequently, a Cox proportional hazards model was employed to investigate the relationship between sarcopenia and depressive symptoms. The follow-up duration started from the baseline assessment in 2011 and continued to the occurrence of the first reported outcome (sarcopenia or depressive symptoms). For participants who did not develop sarcopenia or depressive symptoms during the study period, the follow-up duration was determined from the baseline to the censoring date at the end of the study (2018), the date of death, or to the loss of follow-up.

In order to explore the reciprocal relationship between sarcopenia and depressive symptoms, three models were performed: (1) model 1, adjusted for age and sex; (2) model 2, additionally adjusted for residential region, education level, average household income, BMI, marital status, status of smoking, and alcohol intake; (3) model 3, including comorbidities, sleep duration, and MMSE score. A trend test was conducted by allocating a score value (0, 1, 2) to each sarcopenia status (non-sarcopenia, possible sarcopenia, and sarcopenia) and modeling this value as a continuous variable. The Wald test was then used to determine the statistical significance of this value.

Furthermore, a subgroup analysis was conducted to examine potential variations in the association between sarcopenia and depressive symptoms based on age, gender, sleep duration (≤6 h, >6 h), and physical disease comorbidity. In order to examine effect modification, interaction terms between stratified variables were incorporated. In the sensitivity analysis, participants who disclosed incident outcomes within a two-year period following recruitment were removed. Second, a competing risk model was implemented, which incorporated mortality and loss-of-follow-up as competing events, given that the participants in that model might subsequently develop outcomes.

Participants from the prospective cohort analysis who had comprehensive data on both depressive symptoms and sarcopenia at baseline (time 1) and who also completed at least one follow-up assessment were included in the cross-lagged path analysis. At the follow-up (time 2), depressive symptoms and sarcopenia were assessed using data collected from the participant with the longest duration of complete follow-up. Before conducting the cross-lagged path analysis, regression residual analyses were employed to modify the baseline and subsequent values of depressive symptoms and sarcopenia status for a set of covariates. Subsequently, the values were standardized using Z-transformation (mean 0, SD 1). Coefficients that were standardized were documented for each model. Age, gender, location of residence, marital status, level of education, smoking, and alcohol consumption have been adjusted for in all models. To demonstrate that the models were well-fitting, a comparative fit index (CFI) greater than 0.90 and a standardized root-mean-square residual (SRMR) less than 0.05 were used [43]. Analyses were conducted using SAS software version 9.4 and R version 4.0.2, with a *p* value < 0.05 indicating statistical significance.

## 3. Results

**Figure 1:**
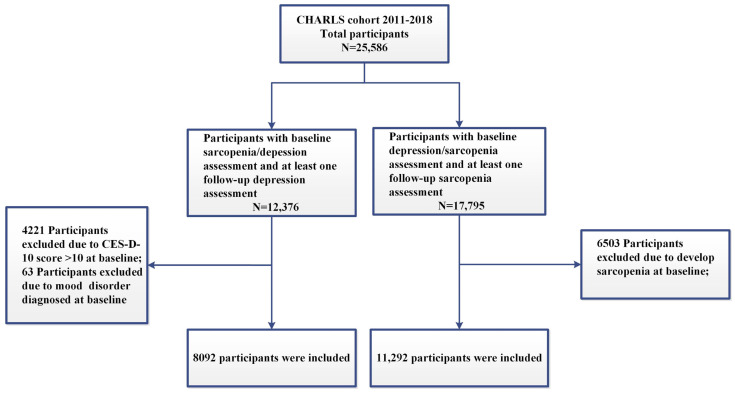
Flow chart of the study population. CHARLS: China Health and Retirement Longitudinal Study; CES-D-10: 10-item Center for Epidemiologic Studies Depressive Scale.

A total of 25,586 participants were interviewed in CHARLS 2011. Subsets of 13,210 and 7791 participants were excluded from the analysis due to the absence of data pertaining to the assessment of sarcopenia or depressive symptoms at both the baseline and follow-up for the two cohorts. In the longitudinal analysis, an additional 4284 participants with a mood disorder diagnosed by a psychiatrist at baseline were excluded from the depressive symptoms cohort, and 6503 participants with sarcopenia at baseline were excluded from the sarcopenia cohort. Our final analytic sample consisted of 8092 and 11,292 participants for the two prospective cohorts, respectively (Figure 1).

### 3.1. Prospective Effect of Sarcopenia Status on the Onset of Depressive Symptoms

Among 8092 participants (median age 57.0, 51.0–64.0), 54.1% were male. Overall, 2379 (29.4%) and 631 (7.9%) were possible sarcopenia and sarcopenia at baseline. There were 1205 (27.5%) cases of possible sarcopenia and 307 (7.0%) cases of sarcopenia in male participants, whereas there were 1174 (31.6%) cases of possible sarcopenia and 324 (8.7%) cases of sarcopenia in female participants. Participants with possible sarcopenia and sarcopenia were more likely to be female, live in a rural area, have a lower education level, have a lower level of household income, be divorced or separated, and have a lower BMI and lower cognitive score (Table 1 and Table A2). The median follow-up duration of this study was 7.0 (interquartile range: 5.0–7.0) years.

**Figure 2:**
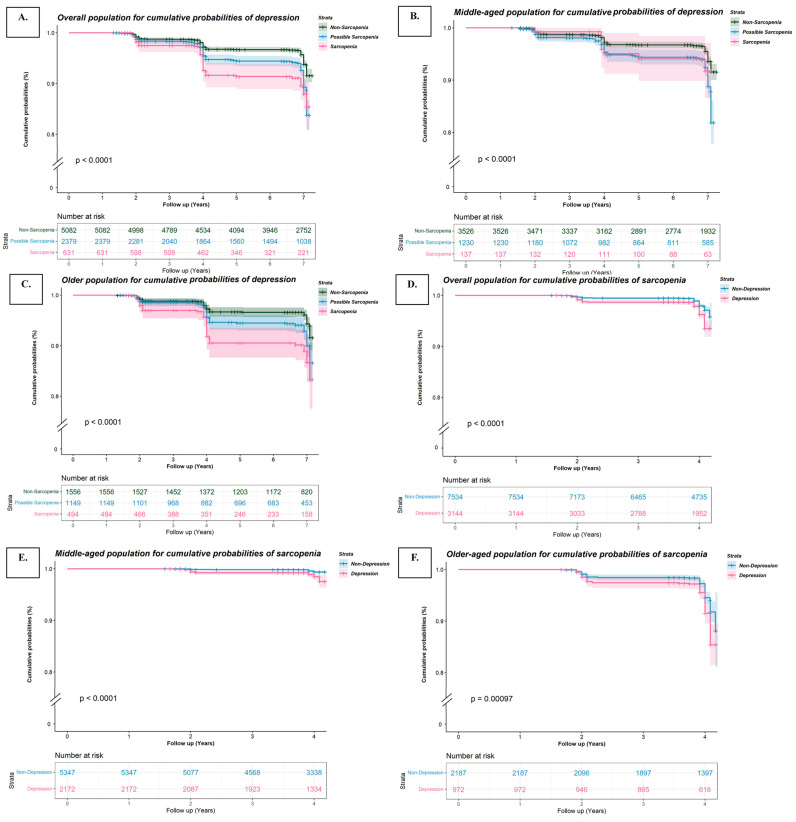
Survival curves of cumulative probabilities of outcomes. (**A**) Overall population for depressive symptoms. (**B**) Middle-aged population for depressive symptoms. (**C**) Old population for depressive symptoms. (**D**) Overall population for sarcopenia. (**E**) Middle-aged population for sarcopenia. (**F**) Old population for sarcopenia.

During a total of 48,305.1 person-years follow-up, 1262 cases of incident depressive symptoms were identified. The 7-year cumulative incidence of depressive symptoms was 15.6% (95%CI: 12.9–18.2) in the possible sarcopenia group and 14.3% (95%CI: 10.0–18.7) in the sarcopenia group versus 8.3% (95%CI: 7.0–9.5) in the non-sarcopenia group (Figure 2A). Possible sarcopenia (HR = 1.5, 95%CI: 1.2–1.8) and sarcopenia (HR = 1.7, 95%CI: 1.2–2.3) are significantly associated with a higher risk of developing depressive symptoms after multivariable adjustment (*p*_trend_ < 0.001, Table 2). Moreover, in the older population, a total of 191 cases developed incident depressive symptoms during 16,466.3 person-years of follow-up. The 7-year cumulative incidence of depressive symptoms was 12.4% (95%CI: 8.9–15.9) in the possible sarcopenia group and 15.2% (95%CI: 10.1–20.4) in the sarcopenia group versus 8.7% (95%CI: 6.2–11.1) in the non-sarcopenia group (Figure 2B). Compared with non-sarcopenia individuals, those with possible sarcopenia and sarcopenia participants showed 1.3- (95%CI: 1.0–1.9) and 1.9-fold (95%CI: 1.3–2.9) higher risk of incident depressive symptoms (*p*_trend_ = 0.0016, Table 2). Among middle-aged participants, 312 incident depressive symptoms cases occurred during 30,006.9 person-years’ follow-up. The 7-year cumulative incidence of depressive symptoms was 8.3% (95%CI: 3.0–13.5) in sarcopenia, 17.4% (95%CI: 13.6–21.1) in possible sarcopenia, and 8.3% (95%CI: 6.8–9.7) in the non-sarcopenia group (Figure 2C). Compared with non-sarcopenia, possible sarcopenia participants were associated with a 1.5-fold (95%CI: 1.2–2.0) higher risk of incident depressive symptoms (Table 2).

### 3.2. Prospective Relationship between Depressive Symptoms and Sarcopenia

The prospective cohort sample consisted of 11,292 participants who had no sarcopenia at baseline and who had at least one follow-up sarcopenia assessment (Figure 1). Among 11,292 participants (median age 55.0, 49.0–61.0), 52.67% were male. Overall, 3277 (29.0%) reported depressive symptoms at baseline. There were 1389 (23.36%) cases of depressive symptoms in male participants and 1888 (35.32%) cases of depressive symptoms in female. Participants with depressive symptoms were more likely to be female, live in a rural area, have a lower education level, have a medium level of household income, be non-married, and have a lower BMI and lower cognitive score (Table 1). The median follow-up duration of this study was 4.0 (interquartile range: 3.9–4.0) years.

During a total of 39,621.1 person-years follow-up, 240 cases of incident sarcopenia were identified. The 4-year cumulative incidence of sarcopenia was 6.5% (95%CI: 4.9–8.2) in the depressive symptoms group versus 2.9% (95%CI: 2.3–3.5) in the non-depressive symptoms group (*p* < 0.001, Figure 2D). Depressive symptoms (HR = 1.5, 95%CI: 1.2–2.0) were significantly associated with a higher risk of developing sarcopenia after multivariable adjustment (*p* < 0.001, Table 3). In the older population, a total of 183 cases developed incident sarcopenia symptoms during 11,781.8 person-years follow-up. The 4-year cumulative incidence of sarcopenia was 14.6% (95%CI: 10.7–18.6) in the depressive symptoms group versus 8.3% (95%CI: 6.3–10.2) in the non-depressive symptoms group (*p* = 0.0010, Figure 2F). Compared with non-depressive symptoms individuals, depressive symptoms participants showed a 1.4-fold (95%CI: 1.0–1.9) higher risk of incident sarcopenia (*p* = 0.0409, Table 3). Among middle-aged participants, 57 incident sarcopenia cases occurred during 27,839.3 person-years’ follow-up. The 4-year cumulative incidence of sarcopenia was 2.5% (95%CI: 1.3–3.6) in the depressive symptoms group and 0.6% (95%CI: 0.4–0.9) in the non-depressive symptoms group (*p* < 0.001, Figure 2E). Compared with non-depressive symptoms, depressive symptoms participants were associated with a 1.5-fold (95%CI: 1.2–2.0) higher risk of incident sarcopenia (Table 4).

### 3.3. Subgroup Analysis

**Figure 3:**
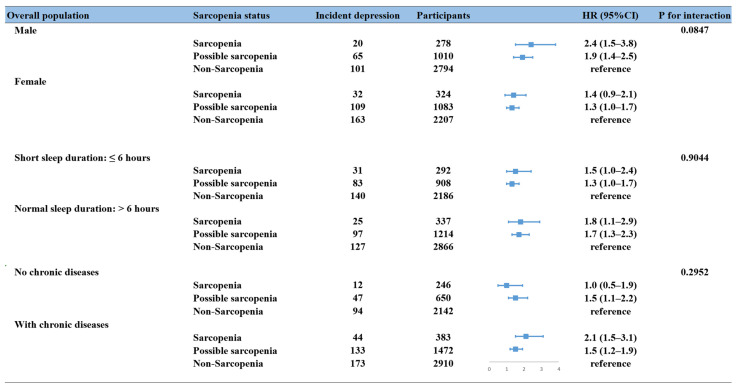
Association between sarcopenia status and depressive symptoms by sex, age, sleep duration, and physical comorbidities. All HRs were calculated by adjusting age, sex, residential region, education level, average household income, BMI, marital status, smoking status, alcohol intake, physical comorbidities, sleep duration, and MMSE score. CI, confidence interval; HR, hazard ratio.

The higher risk of depressive symptoms associated with possible sarcopenia and sarcopenia was observed in overall and in older participants; depressive symptoms were not associated with sarcopenia in the middle-aged group but were significantly associated with possible sarcopenia in this age group (Figure 3). Sarcopenia and possible sarcopenia were significantly associated with an increased risk of depressive symptoms in individuals who were male and older and had more than one type of physical illness. Female, middle-aged participants and participants without a physical illness were more likely to have possible sarcopenia than sarcopenia (Figure 3, Table 2 and Table A3). Independently of participants’ sleep duration, depressive symptoms were associated with sarcopenia and possible sarcopenia.

**Figure 4:**
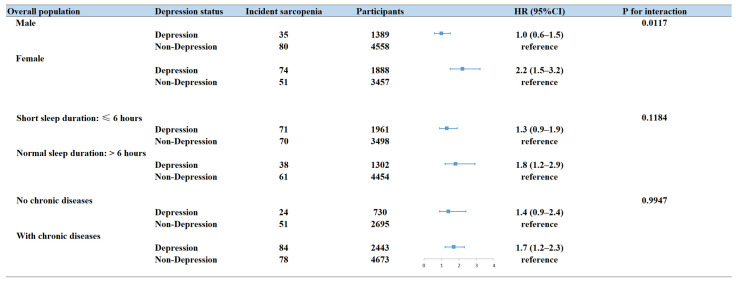
Association between depressive symptoms status and incident sarcopenia by sex, sleep duration, and physical comorbidities. All HRs were calculated by adjusting age, sex, residential region, education level, average household income, BMI, marital status, smoking status, alcohol intake, physical comorbidities, sleep duration, and MMSE score. CI, confidence interval; HR, hazard ratio.

On the other hand, the increased risk of sarcopenia associated with depressive symptoms was consistently observed across various subgroups, including age, sex, sleep duration, and comorbidities (Table 3, Figure 4). Furthermore, significant interaction across sex and depressive symptoms and was observed, with a higher risk of sarcopenia in females (HR = 1.2, 95%CI: 1.5–3.2, Figure 4).

### 3.4. Sensitivity Analysis

**Figure A1:**
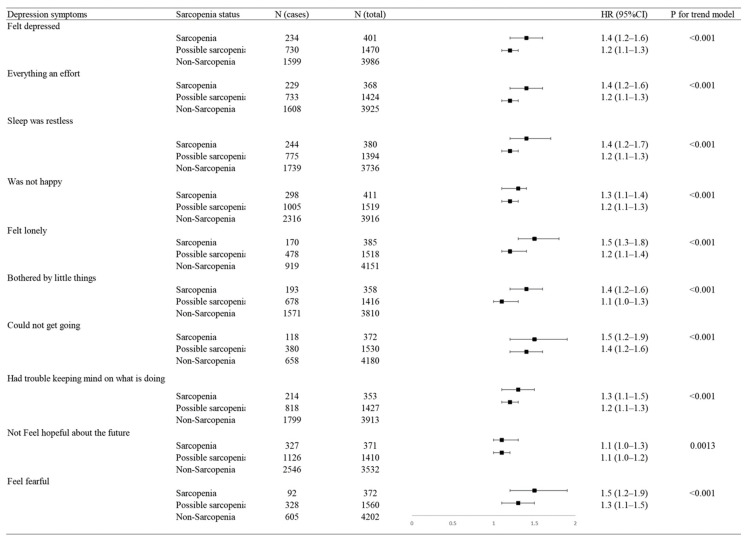
Association between sarcopenia status and each single depressive symptom.

**Figure A2:**
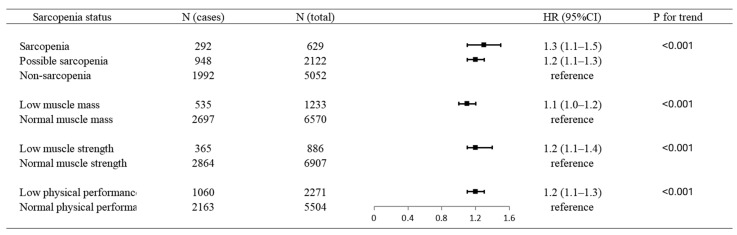
Association between sarcopenia indicators and depressive symptoms.

Results of sensitivity analysis by each single status of sarcopenia and each specific depressive symptoms were similar to primary findings (Figure A1 and Figure A2). Sensitivity analysis excluding incident depressive symptoms cases within 2 years after baseline by performing a competing risk model and a further adjustment of major chronic physical diseases did not markedly affect the results, which indicated the robustness of our results (Table A4).

### 3.5. Bidirectional Association of Depressive Symptoms and Sarcopenia

**Figure 5:**
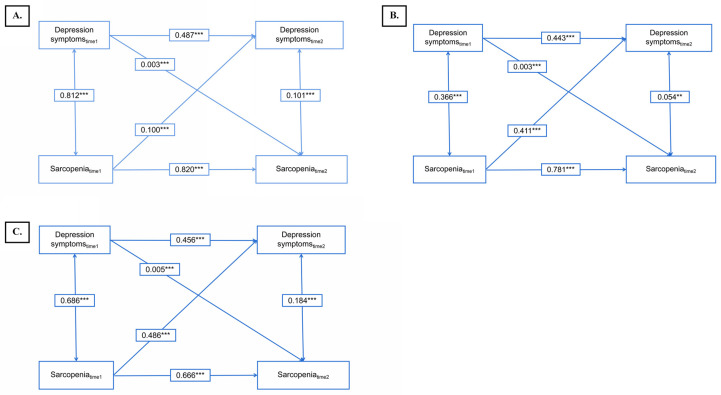
Cross-lagged panel models applied to assess depression symptoms and sarcopenia status, and mediating effect of healthy lifestyle among Chinese adults. (**A**) In the overall population; (**B**) in the middle-aged population; (**C**) in the older-aged population. ** means *p* < 0.01, *** means *p* < 0.001.

Figure 5A illustrates the cross-lagged model exploring the bidirectional relationship between depressive symptoms and sarcopenia (model 1). After controlling for covariates, model 1 fitted the data adequately (CFI = 0.999; SRMR = 0.034). Depressive symptoms at time point 1 were positively related to themselves over time, as was sarcopenia. Furthermore, significant cross-lagged effects emerged, with preceding depressive symptoms significantly predicting sarcopenia at T2 (β = 0.003; *p* < 0.001) and vice versa, with sarcopenia predicting subsequent depressive symptoms at T2 (β = 0.428; *p* < 0.001). The standardized path coefficient from sarcopenia to depressive symptoms was significantly larger than that of the reverse pathway (χ^2^ = 28.516, *p* for difference < 0.001). These findings provide empirical support for our hypothesis positing a bidirectional relationship between depressive symptoms and sarcopenia.

Figure 5B delineates the cross-lagged path analysis examining the interplay between sarcopenia and depressive symptoms within a middle-aged population (model 2). Notably, a significant cross-lagged pathway was observed. Specifically, the standardized path coefficient from sarcopenia at time 1 to depressive symptoms at time 2 (β = 0.411; *p* < 0.001) exhibited notable strength compared to the corresponding pathway from depressive symptoms at time 1 to sarcopenia at time 2 (β = 0.003; *p* < 0.001; χ^2^ = 13.201, *p* for difference = 0.0002798). The fitting indicators (CFI = 0.999 and SRMR = 0.030) underscored the favorable model fit. Figure 5C illustrates the cross-lagged path analysis of sarcopenia and depressive symptoms in the older population (model 3). The cross-lagged pathway was evident, and the standardized path coefficient from sarcopenia at time 1 to depressive symptoms at time 2 (β = 0.486; *p* < 0.001) was markedly stronger than that from depressive symptoms at time 1 to sarcopenia at time 2 (β = 0.005; *p* < 0.001; *χ*^2^ = 18.936, *p* for difference < 0.001). Fitting indicators (CFI = 0.995 and SRMR = 0.048) suggested model 3 to be a good model fit.

## 4. Discussion

This study aimed to examine the bidirectional association between sarcopenia and depressive symptoms using data from a large Chinese national community-based prospective cohort study with an extensive long-term follow-up. Throughout the seven-year follow-up period, a longitudinal bidirectional association was observed between sarcopenia status and depressive symptoms among Chinese middle-aged and older adults. Our findings revealed a 50% and 70% elevated risk of depressive symptoms among participants with possible sarcopenia and sarcopenia, respectively, along with a 50% increased risk of sarcopenia in individuals experiencing depressive symptoms at the outset of the study. The increased risk of depressive symptoms was significantly identified in male, older participants with sarcopenia, whereas sarcopenia risk was higher in female and middle-aged individuals who experienced depressive symptoms. In addition, an examination of standardized cross-lagged coefficients indicated that the temporal connection between initial depressive symptoms and sarcopenia may be more pronounced than the inverse relationship. These results highlight the intricate interplay between sarcopenia and depressive symptoms, underscoring the importance of considering both conditions in comprehensive health management strategies for high-risk adults.

Our study provided new insights into the nature of the relationship between sarcopenia and depressive symptoms. To the best of our knowledge, there have been no studies elucidating the longitudinal reciprocal association between sarcopenia and depression. Similar to cross-sectional studies and meta-analysis, we also found a significant association between depression and sarcopenia [17,18]. However, longitudinal research may offer more robust insights regarding the temporal direction of the observed associations. Recently, two observational studies reported a longitudinal effect of depression on sarcopenia onset [20,44] utilizing two-sample Mendelian randomization and reported evidence suggesting that depression symptoms could potentially result in a decrease in appendicular skeletal muscle mass but not vice versa [20]. In contrast to previous studies, our study not only identified depressive symptoms as a precursor to sarcopenia but also uncovered a notably stronger temporal sequence effect of sarcopenia leading to depressive symptoms. Importantly, this finding held true for both middle-aged and older adults. In terms of sex disparities, while males comprised a majority of the China community population due to socioeconomic and traditional cultural factors, females were more susceptible to developing sarcopenia and depressive symptoms, which is consistent with previous studies [44,45,46].

Diabetes, metabolic syndrome, cardiovascular diseases, and physical inactivity, all of which are associated with an increased risk of depression, have been linked to sarcopenia [47,48]. Neurotrophins, chronic inflammation, and oxidative stress might play a role in the shared pathophysiological mechanisms bridging sarcopenia and depressive symptoms [49,50,51]. Previous studies had shown that decreased muscle mass and strength have significant immune and redox effects by affecting the expression of neurotrophins (such as BDNF) and pro-inflammatory cytokines (CRP, IL-6, TNF-α), which consequently affect the development of depressive symptoms [51,52,53,54,55]. Another plausible explanation might be that the loss of skeletal muscle mass and function leads to less physical activity and malnutrition, which accordingly increases the risk of occurrence of depressive symptoms [27,56].

Increased evidence has demonstrated that a lack of physical activity and lower nutrition intake (e.g., protein, fruit, vegetables, and vitamin D) are associated with the upregulation of chronic inflammation and oxidative stress, which in turn lead to depressive symptoms and sarcopenia [57,58,59,60,61,62,63]. To date, several feasible interventions used to improve sarcopenia have been proven effective in older adults, such as resistance training, protein supplement, and pharmacologic intervention [64,65,66,67]. Given the absence of primary prevention guidelines for sarcopenia in China as of yet, implementing early depression screening in community-based care services and encouraging individuals to maintain a healthy lifestyle could aid in the identification of those at increased risk of developing sarcopenia [44]. Assessing sarcopenia in male and older participants may facilitate the early detection and treatment of depressive symptoms and, accordingly, could reduce the incidence of major depressive symptoms disorder. While this study may have focused on sarcopenia and depression prevention in China, its implications extend to populations globally. By advocating for the adoption of healthy lifestyle practices and the implementation of early screening protocols for depression, this discovery enriches the broader discourse on proactive strategies aimed at mitigating sarcopenia, combating depression, and fostering healthy aging on a global scale.

While our study highlighted the stronger directional influence of early sarcopenia on subsequent depressive symptoms compared to the reverse pathway, we acknowledge the significance of addressing the impact of depressive symptoms on sarcopenia. Our findings suggest that early screening for depressive symptoms may also be beneficial in mitigating the occurrence of sarcopenia, particularly among females and middle-aged individuals. Given that both sarcopenia and depressive symptoms could independently predict cardiovascular disease and mortality, the early identification and intervention of these conditions may be helpful to prevent overall poor outcomes [10,30,68].

This research possesses several strengths. To begin with, individuals who were 45 years of age or older were selected as participants from the CHARLS study, a population-based prospective cohort that is nationally representative. In this study, well-validated measures were used to assess depressive symptoms and sarcopenia status. Furthermore, to clarify the temporal bidirectional relationships between these two factors, our investigation employed a cross-lagged panel model, compared to other prospective studies that followed a unidirectional approach. Finally, it is important to note that this research is the first endeavor to investigate the possible temporal progression of alterations in depressive symptoms and sarcopenia in the Chinese population. As such, our results merit additional verification and investigation.

Potential limitations also need to be addressed. First, the research was an observational study, and recall bias in questionnaire surveys was unavoidable. Furthermore, it should be noted that although the statistical significance of the reciprocal associations between depressive symptoms and sarcopenia was preserved even after controlling for numerous potential confounding variables, there might be additional confounding factors that were not accounted for in our research. Additional potential confounding variables, including but not limited to dietary intake, body fat mass, and other psychiatric disorders like eating disorders and substance use disorder, as well as inflammation factors such as IL-6 and TNF-α, should be considered in future research. Third, it is important to highlight that the evaluation of gait speed was conducted at a distance of 2.5 m, which deviates from the traditional 6 m standard. However, an earlier investigation indicated that the gait speed measurements acquired by CHARLS were consistent with those obtained in China, suggesting that the distance discrepancy did not have an effect on the gait speed results [68]. Fourth, the data utilized spanned from 2011 to 2018, potentially limiting its relevance to current circumstances. Factors such as the COVID-19 pandemic in 2020 could have influenced the prevalence of both sarcopenia and depressive symptoms, necessitating updated data to accurately depict the current state of conditions. Finally, a significant proportion of individuals lacked initial or subsequent assessments for depressive symptoms and sarcopenia; this incomplete follow-up may introduce potential bias into our findings, despite the substantial number of eligible participants involved in the study.

## 5. Conclusions

In conclusion, this study identified a longitudinal reciprocal association between sarcopenia and depressive symptoms. Individuals who are male and older than 60 are more sensitivity to sarcopenia and develop an increased risk of depressive symptoms in later life. Female and middle-aged adults are vulnerable to depressive symptoms and exhibited a higher likelihood to develop sarcopenia. Cross-lagged path coefficients suggest that the association between initial sarcopenia and subsequent depressive symptoms is more likely to be significant than the reverse pathway. The early assessment and improvement of sarcopenia and depressive symptoms may be beneficial to reduce the risk of poor overall health and foster the process of healthy aging within the Asian population. Further research is necessary to unravel this relationship and elucidate the underlying mechanism. Given the established association between sarcopenia and depressive symptoms, it is imperative to develop further cost-effective therapies.

## Figures and Tables

**Table 1:** Characteristics of all participants at baseline by sarcopenia.

Variables	Total (N = 8092)	Non-Sarcopenia (N = 5082)	Possible Sarcopenia (N = 2379)	Sarcopenia (N = 631)	*p*-Value
Age (Median, Q_25_–Q_75_)	57.0 (51.0–64.0)	55.0 (49.0–61.0)	59.0 (53.0–66.0)	68.0 (61.0–75.0)	<0.001
Gender					
Male	4375 (54.07)	2863 (56.34)	1205 (50.65)	307 (48.65)	<0.001
Female	3717 (45.93)	2219 (43.66)	1174 (49.35)	324 (51.35)	
Residential area					<0.001
Urban	3278 (40.51)	2069 (40.71)	1024 (43.04)	185 (29.32)	
Rural	4814 (59.49)	3013 (59.29)	1355 (56.96)	446 (70.68)	
Education					<0.001
Illiterate	1818 (22.47)	905 (17.81)	640 (26.9)	273 (43.26)	
Primary school	3182 (39.32)	1984 (39.04)	953 (40.06)	245 (38.83)	
Middle school	1904 (23.53)	1357 (26.7)	479 (20.13)	68 (10.78)	
High school/vocational high school	1018 (12.58)	725 (14.27)	252 (10.59)	41 (6.5)	
Junior college or above	170 (2.1)	111 (2.18)	55 (2.31)	4 (0.63)	
Average household income (CNY)					<0.001
<1000	3978 (49.16)	2590 (50.96)	1114 (46.83)	274 (43.42)	
1000	810 (10.01)	436 (8.58)	279 (11.73)	95 (15.06)	
5000	492 (6.08)	291 (5.73)	153 (6.43)	48 (7.61)	
10,000	691 (8.54)	408 (8.03)	209 (8.79)	74 (11.73)	
20,000	2121 (26.21)	1357 (26.7)	624 (26.23)	140 (22.19)	
Marital status					
Married	6749 (83.4)	4430 (87.17)	1889 (79.4)	430 (68.15)	<0.001
Separated	359 (4.44)	221 (4.35)	124 (5.21)	14 (2.22)	
Unmarried/divorced/widowed	984 (12.16)	431 (8.48)	366 (15.38)	187 (29.64)	
Smoking status					<0.001
No	4170 (51.54)	2540 (49.99)	1305 (54.85)	325 (51.51)	
Yes	2405 (29.72)	1614 (31.77)	612 (25.73)	179 (28.37)	
Previous	1516 (18.74)	927 (18.24)	462 (19.42)	127 (20.13)	
Alcohol drinking					<0.001
No	4177 (51.62)	2536 (49.9)	1294 (54.39)	347 (54.99)	
Yes	2984 (36.88)	2043 (40.2)	747 (31.4)	194 (30.74)	
Previous	931 (11.51)	503 (9.9)	338 (14.21)	90 (14.26)	
Daily sleep time (hours)	7.00 (6.00–8.00)	7.00 (6.00–8.00)	7.00 (6.00–8.00)	7.00 (5.00–8.00)	0.151
MMSE score, (Median, Q_25_–Q_75_)	16.0 (12.0–18.0)	16.00 (13.00–19.00)	15.00 (12.00–18.00)	14.00 (10.00–15.00)	<0.001
BMI (kg/m^2^; Median, Q_25_–Q_75_)	23.4 (21.1–26.0)	23.61 (21.37–26.05)	24.25 (22.25–26.80)	19.08 (18.00–20.14)	<0.001
Sarcopenia indicators					
Low muscle mass	1244 (15.85)	610 (12)	3 (0.14)	631 (100)	<0.001
Low muscle strength	908 (11.56)	0 (0)	614 (28.64)	294 (46.67)	<0.001
Low physical performance	2284 (29.18)	0 (0)	1775 (83.81)	509 (81.18)	<0.001

CNY, Chinese Yuan; MMSE, Mini-Mental State Examination; BMI, body mass index.

**Table 2:** Risk of depression associated with baseline sarcopenia status.

	Non-Sarcopenia	Possible Sarcopenia	Sarcopenia	*p* for Trend
Overall population (N = 8092)				
No. of participants	5082	2379	631	
No. of incident depression	267	193	56	
Follow-up, person-years	31,708.70	13,332.50	3263.90	
Follow-up, years Median (IQR)	7.0 (6.9–7.0)	6.9 (4.0–7.0)	6.9 (4.0–7.0)	
Hazard ratio for incident depression (95%CI, *p* value)				
Unadjusted	reference	1.8 (1.5–2.1)	2.1 (1.6–2.9)	<0.001
Adjusted model 1	reference	1.7 (1.4–2.1)	2.0 (1.5–2.8)	<0.001
Adjusted model 2	reference	1.6 (1.4–2.0)	1.8 (1.3–2.4)	<0.001
Adjusted model 3	reference	1.5 (1.2–1.8)	1.7 (1.2–2.3)	<0.001
Older adults, age ≥ 60 (N = 2910)				
No. of participants	1389	1047	474	
No. of incident depression	74	73	44	
Follow-up, person-years	8469.60	5653.70	2343	
Follow-up, years Median (IQR)	7.0 (5.0–7.0)	7.0 (4.0–7.0)	4.0 (4.0–7.0)	
Hazard ratio for incident depression (95%CI, *p* value)				
Unadjusted	reference	1.5 (1.1–2.1)	2.3 (1.6–3.3)	<0.001
Adjusted model 1	reference	1.5 (1.1–2.1)	2.3 (1.5–3.4)	<0.001
Adjusted model 2	reference	1.4 (1.0–1.9)	1.9 (1.3–2.9)	<0.001
Adjusted model 3	reference	1.3 (1.0–1.9)	1.9 (1.3–2.9)	0.0016
Middle-aged adults, age < 60 (N = 4893)				
No. of participants	3526	1230	137	
No. of incident depression	191	112	9	
Follow-up, person-years	22,154.20	7060.80	791.9	
Follow-up, years Median (IQR)	7.0 (6.9–7.0)	7.0 (4.0–7.0)	7.0 (4.0–7.0)	
Hazard ratio for incident depression (95%CI, *p* value)				
Unadjusted	reference	1.8 (1.5–2.3)	1.3 (0.7–2.6)	<0.001
Adjusted model 1	reference	1.8 (1.4–2.2)	1.2 (0.6–2.4)	<0.001
Adjusted model 2	reference	1.7 (1.3–2.1)	1.0 (0.5–2.0)	<0.001
Adjusted model 3	reference	1.5 (1.2–2.0)	1.0 (0.5–2.0)	0.0114

No. of participants, number of participants; IQR, interquartile range; 95%CI, 95% confidence interval.

**Table 3:** Characteristics of all participants at baseline by depressive symptoms.

Variables	Total (N = 11,292)	Non-Depression (N = 8015)	Depression (N = 3277)	*p*-Value
Age (Median, Q_25_–Q_75_)	55.0 (49.0–61.0)	55.0 (49.0–61.0)	56.0 (49.0–61.0)	<0.001
Gender				
Male	5947 (52.67)	4558 (56.87)	1389 (42.39)	<0.001
Female	5345 (47.33)	3457 (43.13)	1888 (57.61)	
Residential area				
Urban	3787 (40.49)	2850 (43.8)	937 (32.93)	<0.001
Rural	5565 (59.51)	3657 (56.2)	1908 (67.07)	
Education				
Illiterate	2267 (20.08)	1382 (17.24)	885 (27.01)	<0.001
Primary school	4608 (40.81)	3142 (39.2)	1466 (44.75)	
Middle school	2697 (23.89)	2052 (25.6)	645 (19.69)	
High school/vocational high school	1445 (12.8)	1193 (14.88)	252 (7.69)	
Junior college or above	274 (2.43)	246 (3.07)	28 (0.85)	
Average household income (CNY)				
<1000	6757 (59.84)	4969 (62)	1788 (54.56)	<0.001
1000–5000	883 (7.82)	506 (6.31)	377 (11.5)	
5000–10,000	584 (5.17)	356 (4.44)	228 (6.96)	
10,000–20,000	763 (6.76)	488 (6.09)	275 (8.39)	
>20,000	2305 (20.41)	1696 (21.16)	609 (18.58)	
Marital status				
Married	9494 (84.08)	6911 (86.23)	2583 (78.82)	<0.001
Separated	644 (5.7)	422 (5.27)	222 (6.77)	
Unmarried/divorced/widowed	1154 (10.22)	682 (8.51)	472 (14.4)	
Never/current smoker				
No	5941 (52.64)	4037 (50.39)	1904 (58.14)	<0.001
Yes	3493 (30.95)	2569 (32.07)	924 (28.21)	
Never smoked	1852 (16.41)	1405 (17.54)	447 (13.65)	
Never/current alcohol				
No	5726 (50.71)	3915 (48.85)	1811 (55.26)	<0.001
Yes	4361 (38.62)	3313 (41.33)	1048 (31.98)	
Never drank alcohol	1205 (10.67)	787 (9.82)	418 (12.76)	
Daily sleep time (hours)	7.00 (5.00–8.00)	7.00 (6.00–8.00)	6.00 (5.00–7.00)	<0.001
MMSE score, (Median, Q_25_–Q_75_)	16.00 (13.00–18.00)	16.00 (13.00–19.00)	15.00 (12.00–16.00)	<0.001
BMI (kg/m^2^; Median, Q_25_–Q_75_)	24.2 (21.3–26.0)	23.8 (21.5–26.2)	22.9 (20.7–25.5)	<0.001

CNY, Chinese Yuan; MMSE, Mini-Mental State Examination; BMI, body mass index; Q_25_, 25th percentile; Q_75_, 75th percentile.

**Table 4:** Risk of sarcopenia associated with baseline depression.

	Non-Depression	Depression	*p* Value
Overall population (N = 11,292)			
No. of participants	8015	3277	
No. of incident depression	131	109	
Follow-up, person-years	27,833.5	11,787.6	
Follow-up, years Median (IQR)	4.0 (3.9–4.0)	4.0 (3.9–4.0)	
Hazard ratio for incident depression (95%CI, *p* value)			
Unadjusted	reference	2.0 (1.5–2.5)	<0.001
Adjusted model 1	reference	1.8 (1.4–2.4)	<0.001
Adjusted model 2	reference	1.6 (1.3–2.1)	<0.001
Adjusted model 3	reference	1.5 (1.2–2.0)	0.0032
Older adults, age ≥ 60 (N = 3303)			
No. of participants	2293	1010	
No. of incident depression	105	78	
Follow-up, person-years	8127.47	3654.31	
Follow-up, years Median (IQR)	4.0 (3.9–4.0)	4.0 (3.9–4.0)	
Hazard ratio for incident depression (95%CI, *p* value)			
Unadjusted	reference	1.6 (1.2–2.2)	0.0012
Adjusted model 1	reference	1.6 (1.2–2.1)	0.0022
Adjusted model 2	reference	1.5 (1.1–2.0)	0.0164
Adjusted model 3	reference	1.4 (1.0–1.9)	0.0409
Middle-aged adults, age < 60 (N = 7989)			
No. of participants	5722	2267	
No. of incident sarcopenia	26	31	
Follow-up, person-years	19,705.9	8133.3	
Follow-up, years Median (IQR)	4.0 (3.9–4.0)	4.0 (3.9–4.0)	
Hazard ratio for incident depression (95%CI, *p* value)			
Unadjusted	reference	2.9 (1.7–4.9)	<0.001
Adjusted model 1	reference	2.7 (1.6–4.5)	<0.001
Adjusted model 2	reference	2.4 (1.4–4.2)	0.0021
Adjusted model 3	reference	2.2 (1.3–4.0)	0.0059

IQR, interquartile range; 95%CI, 95% confidence interval.

## Data Availability

The China Health and Retirement Longitudinal Study (CHARLS) data set is publicly available at http://charls.pku.edu.cn/en (accessed on 27 May 2023). The data analyzed in our study can be obtained from the corresponding author upon request.

## Appendix A

**Table A1:** Inclusion and exclusion criteria for the two prospective cohorts.

Cohort 1: Depressive Symptoms Cohort		Cohort 2: Sarcopenia Cohort	
Inclusion Criteria	Exclusion Criteria	Inclusion Criteria	Exclusion Criteria
Individuals aged 45 years or older in 2011	Missing data on all depressive symptoms in 2013, 2015, or 2018	Individuals aged 45 years or older in 2011	Absence of data pertaining to all components associated with sarcopenia in the years 2013, 2015, or 2018
Possessing baseline data concerning both sarcopenia status	Absence of data pertaining to all demographic variables and covariates	Individuals who had baseline data on the assessment of depressive symptoms in 2011, either through participation in the CES-D-10 evaluation or by reporting a diagnosis of mood disorders by doctors or taking medication for mood disorders.	Participants who had sarcopenia at baseline
Individuals who neither received a diagnosis of mood disorders from doctors nor took any medication to treat emotional, nervous, or mental problems, and those with CES-D-10 scores ≤ 10 at baseline			Lack of information concerning demographic variables and covariates

**Table A2:** Chronic diseases of all participants at baseline by sarcopenia.

Variables	Total (N = 8092)	Non-Sarcopenia (N = 5082)	Possible Sarcopenia (N = 2379)	Sarcopenia (N = 631)	*p*-Value
Co-morbidities					
Cancer	49 (0.61)	27 (0.53)	18 (0.76)	4 (0.63)	0.5017
Chronic lung disease	621 (7.68)	317 (6.24)	212 (8.92)	92 (14.58)	<0.001
Heart disease	756 (9.34)	402 (7.91)	302 (12.7)	52 (8.24)	<0.001
Stroke	131 (1.62)	55 (1.08)	65 (2.73)	11 (1.74)	<0.001
Arthritis	2088 (25.81)	1203 (23.67)	727 (30.57)	158 (25.04)	<0.001
Dyslipidaemia	663 (8.21)	413 (8.13)	236 (9.96)	14 (2.22)	<0.001
Hepatic disease	190 (2.35)	112 (2.21)	65 (2.74)	13 (2.06)	0.3275
Kidney disease	303 (3.75)	181 (3.56)	100 (4.21)	22 (3.49)	0.3689
Digestive system disease	1340 (16.56)	828 (16.3)	389 (16.36)	123 (19.49)	0.1192
Asthma	520 (6.45)	260 (5.14)	185 (7.8)	75 (11.94)	<0.001
Memory-related disease	43 (0.53)	12 (0.24)	26 (1.1)	5 (0.8)	<0.001
Hypertension	1929 (23.85)	1056 (20.79)	764 (32.13)	109 (17.27)	<0.001
Diabetes	504 (6.23)	295 (5.81)	195 (8.2)	14 (2.22)	<0.001

**Table A3:** Chronic disease of all participants at baseline by depressive symptoms.

Variables	Total (N = 11,292)	Non-Depression (N = 8015)	Depression (N = 3277)	*p*-Value
Co-morbidities				
Cancer	90 (0.8)	60 (0.76)	30 (0.92)	0.3818
Chronic lung disease	920 (8.23)	529 (6.68)	391 (11.99)	<0.001
Heart disease	1138 (10.18)	700 (8.84)	438 (13.45)	<0.001
Stroke	191 (1.71)	108 (1.36)	83 (2.55)	<0.001
Arthritis	3170 (28.33)	1829 (23.07)	1341 (41.11)	<0.001
Dyslipidemia	1055 (9.45)	745 (9.41)	310 (9.54)	0.8354
Hepatic disease	346 (3.1)	202 (2.55)	144 (4.42)	<0.001
Kidney disease	537 (4.8)	300 (3.79)	237 (7.28)	<0.001
Digestive system disease	2280 (20.38)	1321 (16.66)	959 (29.41)	<0.001
Asthma	743 (7.92)	385 (5.92)	358 (12.45)	<0.001
Memory-related disease	55 (0.49)	30 (0.38)	25 (0.77)	0.0073
Hypertension	2570 (22.98)	1788 (22.56)	782 (24.02)	0.0952
Diabetes	791 (7.17)	532 (6.81)	259 (8.05)	0.0224

**Table A4:** Sensitivity analysis regarding risk of depressive symptoms with baseline sarcopenia status.

Sarcopenia Status	Incident Depression	Participants	HR (95%CI)	*p* Value	*p* for Trend
Overall population					
Sensitivity analysis 1: excluding depression participants diagnosed within 2 years after baseline (N = 7352)					<0.001
Sarcopenia	45	542	1.7 (1.2–2.5)	0.0021	
Possible sarcopenia	155	1949	1.5 (1.3–1.9)	<0.001	
Non-Sarcopenia	226	4861	reference		
Sensitivity analysis 2: competing risk model (N = 7803, No. of competing events = 110)					
Sarcopenia	56	629	1.7 (1.3–1.8)	0.0007	<0.001
Possible sarcopenia	179	2122	1.5 (1.2–1.8)	<0.001	
Non-Sarcopenia	264	5052	reference		
